# White‐Matter Structural Connectivity and Alzheimer's Disease: A Mendelian Randomization Study

**DOI:** 10.1002/brb3.70286

**Published:** 2025-02-09

**Authors:** Siyu Liu, Daoying Geng

**Affiliations:** ^1^ Radiology Department, Huashan Hospital Affiliated with Fudan University Shanghai China; ^2^ Shanghai Engineering Research Center of Intelligent Imaging for Critical Brain Diseases Shanghai China; ^3^ Institute of Functional and Molecular Medical Imaging Fudan University Shanghai China

**Keywords:** Alzheimer's disease | genome‐wide association study | Mendelian randomization | Right‐hemisphere limbic network to thalamus white‐matter structural connectivity | white‐matter structural connectivity

## Abstract

**Background:**

Alzheimer's disease (AD) and white‐matter structural connectivity have been linked in some observational studies, although it is unknown if this is a causal relationship. The purpose of this study was to examine the impact of various white‐matter structural connectivity on AD via a two‐sample multivariate Mendelian randomization (MR) approach.

**Methods:**

The genome‐wide association study (GWAS) of Wainberg et al. provided the summary data on white‐matter structural connectivity, and Bellenguez et al.’s study provided the GWAS aggregated data for AD. MR methods included inverse variance weighted, Mendelian randomization Egger, simple mode, weighted median, and weighted mode. Heterogeneity, horizontal pleiotropy, and “leave‐one‐out” analysis guaranteed the robustness of causation. Finally, reverse MR analysis was conducted on the white‐matter structural connectivity that showed positive results in the forward MR analysis.

**Results:**

Among 206 white‐matter structural connections, we identified 10 connections were strongly correlated with genetic susceptibility to AD. Right‐hemisphere limbic network to thalamus white‐matter structural connectivity and Right‐hemisphere salience_ventral attention network to accumbens white‐matter structural connectivity were positively correlated with the likelihood of AD, while the remaining 8 white‐matter structural connections were negatively related with AD. None of the above 10 white‐matter structural connections have a reverse causal relationship with AD.

**Conclusion:**

Our MR study reveals a certain degree of association between white‐matter structural connectivity and AD, which may provide support for future diagnosis and treatment of AD.

## Graphical abstract

This study used a two‐sample bidirectional MR technique to evaluate the most recent summary GWAS data and revealed a causal relationship between white‐matter structural connectivity and AD, which is the first time that the relationship between these two has been investigated at the genetic level.

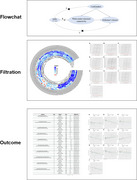

## 1 Introduction

As the problem of ageing becomes more acute in countries around the world, diseases associated with aging are gradually affecting our social life (Carmona and Michan 2016). The most prevalent kind of dementia is Alzheimer's disease (AD), an irreversible primary degenerative disease of the central nervous system (Chandra, Dervenoulas, and Politis 2019). AD occurs in people over 40 and is mainly characterized by progressive cognitive dysfunction and behavioral impairments, with progressive loss of ability to live and eventual death due to multiple complications (Zhang et al. 2023). Current research has found that AD is also the fifth leading cause of death worldwide (GBD 2019 Dementia Forecasting Collaborators 2022). AD patients' careers face heightened financial and psychological strain, rendering the social and familial load of caring for AD patients immense and unmanageable (Alzheimer's Association 2020; Zhang et al. 2021). The etiology of AD has not yet been clarified, so it is essential to look for potential exposures associated with AD in order to enable early diagnosis and intervention.

Neuroscience is developing rapidly in the study of neural data, and these large‐scale datasets represent a diverse network of connections, which include synaptic connections and anatomical projections between brain regions (Bassett and Sporns 2017; Medaglia, Lynall, and Bassett 2015; Sejnowski, Churchland, and Movshon 2014). Traditional studies of human brain anatomy have mostly remained at the macroscopic (lobes, white matter, grey matter) or microscopic (cells, molecules) anatomical level, without more detailed information on brain area connectivity patterns, but only recorded large‐scale network features in the cerebral cortex of some mammals (Ardesch et al. 2022; Scannell et al. 1999; Sporns, Tononi, and Kötter 2005; Stephan et al. 2001). Myelinated axon bundles make up cerebral white matter, which is thought to be a sign of how connected the cortical system is (Harris and Attwell 2012; Zhang and Sejnowski 2000). A brain region is defined by the fact that all its structural constituents have extremely comparable long‐distance connections, which determine the functional characteristics and anatomical properties of the region (Passingham, Stephan, and Kötter 2002). White matter fibers can communicate information across these various brain regions faster thanks to myelin sheaths, which causes modest changes in brain function related to cognition (Schmidt and Knösche 2019).

An epidemiological method called Mendelian randomization (MR) employs observational data to calculate causality (Birney 2022; Sekula et al. 2016). Genome‐wide association studies (GWAS) have revealed a multitude of genetic variations correlated with human diseases in the field of genetic epidemiology (Abdellaoui et al. 2023). The basic idea of MR is to use genetic variants with strong correlations with exposures as instrumental variables (IVs) to infer causal effects between exposures and study outcomes with single nucleotide polymorphisms (SNPs) being the most commonly used IVs, thus eliminating the impact of confounding variables and ethics (Birney 2022; Bowden and Holmes 2019). According to Wainberg et al., AD is genetically associated with white‐matter structural connectivity but does not specify which connections are related to AD risk (Wainberg et al. 2024).

Most past studies have focused on the connections and numerical changes between the macroscopic networks of the whole brain in AD patients. Elsheikh et al. quantified changes in the brain connectome of AD patients through characteristic path length and weighted global efficiency (Elsheikh et al. 2020). They identified significant SNPs and genes associated with AD progression and numerical changes in the brain connectome. However, this was a study conducted at the global brain level and did not involve connections between specific brain regions. Mitra et al. conducted an inter‐brain region differential correlation (inter‐DC) analysis on RNA sequencing data from four macroscopic brain regions (frontal pole, superior temporal gyrus, parahippocampal gyrus, and inferior frontal gyrus) (Mitra et al. 2024). They found that DC genes within this network could complement the known differentially expressed genes in AD, potentially reflecting intrinsic cellular changes and providing a new perspective for AD etiology research. Xiong et al. identified eight genes associated with brain networks based on cortical morphology and found that genetic variations can affect these brain networks (Xiong et al. 2024). Mirza‐Davies et al. employed whole‐brain fiber tractography using diffusion MRI data to create the whole‐brain connectome and structural brain networks for the default mode, limbic, and visual subnetworks in their investigation of the association between structural brain networks in young adults and AD polygenic risk (Mirza‐Davies et al. 2022). They calculated various metrics for these networks for each participant and found that brain connectivity was slightly decreased in young adults with a higher risk of developing AD in later life.

The discoveries made by these researchers regarding the genetic aspects of AD and white‐matter structural connections are greatly helpful to our study. However, the white‐matter structural connections they used were all at the macroscopic level, such as the whole‐brain or gyrus level. Even though Mirza‐Davies et al. used some network connections in their study, they were still limited to that scope. This study employed a two‐sample MR approach for the first time to analyze the association between white‐matter structural connectivity and AD. Compared with previous related research, using MR analysis can comprehensively identify specific white‐matter structural connections. This novel study approach offers the most recent scientific proof and unique contributions for early diagnosis and therapy by enabling a more focused examination of the ways in which white‐matter structural connections impact the development and course of AD.

## 2 Materials and Methods

### 2.1 Design of the Study and Data Source

**FIGURE 1:**
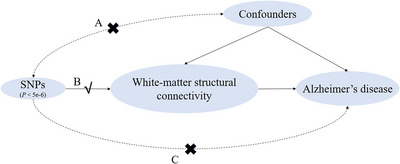
This MR study's flowchart. SNPs are genetic IVs. White‐matter structural connectivity is the exposure factor. Alzheimer's disease is an outcome. (A) Independence assumption: IVs are independent of confounders that affect “exposure and outcome”. (B) Association assumption: There is a strong association assumption between IVs and exposure factor. (C) Exclusion assumption: IVs can only affect the outcome through the exposure factor, not through other pathways. The study completed the causality analysis of the relationship between AD and white‐matter structural connectivity through Pathway B.

Figure 1 illustrates the research design procedure we used. In this study, we used two‐sample MR analysis with white‐matter structural connectivity as exposure and AD as an outcome to screen for eligible SNPs as instrumental variables.

GWAS summary data were derived from the study of Wainberg et al. who performed a genome‐wide association investigation of 206 structural connectivity of brain regions obtained from tractography across 26,333 UK Biobank participants (Wainberg et al. 2024). The structural connectivity used in this study was measured through a technique called fiber tracking or tractography (Basser et al. 2000; Conturo et al. 1999; Jeurissen et al. 2019). Fiber tractography can quantify the structural connectome by superimposing a selected brain atlas of an individual, providing rich, whole‐brain details related to cognition (Medaglia, Lynall, and Bassett 2015; Wainberg et al. 2024). These connections were generalized into three types, including hemisphere‐level (left hemisphere, right hemisphere) cortical‐to‐cortical connectivity (3 measures), network‐level cortical‐to‐cortical connectivity within and between each of the 14 hemisphere‐specific “Yeo 7” networks (SomMot somatomotor, Vis visual, DorsAttn dorsal attention, SalVentAttn salience/ventral attention, Cont control, Limbic limbic, Default default mode) (105 measures) and cortical‐to‐subcortical connectivity between each of these 14 “Yeo 7” networks and 7 subcortical structures (thalamus, caudate, putamen, pallidum, hippocampus, amygdala, accumbens) (98 measures) (Wainberg et al. 2024; Yeo et al. 2011). We investigated these 206 connections to examine the association between AD and white‐matter structural connectivity.

The Bellenguez et al. provided the GWAS aggregated data for AD, which were stored in the European Bioinformatics Institute GWAS Catalog (Bellenguez et al. 2022). AD summary statistics were obtained from the GWAS dataset from 2022 (https://gwas.mrcieu.ac.uk/datasets/ebi‐a‐GCST90027158/), which comprised 46,828 controls and 39,016 cases of European descent. A total of 20,921,626 SNPs were eventually analyzed.

### 2.2 Selection of IVs

To guarantee the dependability of white‐matter structural connectivity and AD and to reduce the impact of bias, the selection of IVs must comply with the three MR assumptions. First, the IVs have to be strongly attached to white‐matter structural connectivity. Second, these SNPs must not be related to other confounders. Finally, all selected SNPs can affect the outcome only through white‐matter structural connectivity and not in other ways. Based on the above assumptions, these criteria were followed to select the most appropriate IVs: (1) The potential IVs were SNPs screened by a level of statistical significance (*P* < 5×10^−6^); (2) To lessen the interaction between similar SNPs, it was necessary to remove all SNPs in linkage disequilibrium (LD) and retain only the most significant SNPs for subsequent analyses (clumping window size  = 10,000 kb, *R* ^2^ < 0.001); (3) When palindrome SNPs were present, it needs to be removed. LD refers to the phenomenon where the probability of two or more allelic genes from different loci appearing simultaneously on a single chromosome is higher than the expected random frequency. The frequency of this combination will deviate from the expected value of random combination, so we need to exclude it. Our study focuses more on discovering new connections between candidate genes and AD. Therefore, when selecting the threshold, we chose a more lenient *P* < 5×10^−6^ as the threshold for screening SNPs in our study, aiming to uncover new associations while maintaining the accuracy of the analysis.

To eliminate weak IVs, the F‐statistic was computed. An F‐statistic of less than 10 indicated that the SNP was a weak instrumental variable. The F‐statistics were calculated using the following formula: *F* = [(*N*−*K*−1)/*K*] ×*R* ^2^/(1−*R* ^2^), where *R* ^2^ represented the proportion of variance in the white‐matter structural connectivity or the percentage of variance in genetic variation explained by selected SNPs, *N* represented the sample size, and *K* represented the number of IVs (Burgess, Butterworth, and Thompson 2013; Pierce, Ahsan, and Vanderweele 2011). *R* ^2^ was calculated as follows:

$$\begin{array}{ccc} & & R^{2}=[\left(1-\mathrm{EAF}\right)\times 2\times \beta^{2}\times \mathrm{EAF}]/[2\times \beta^{2}\times \mathrm{EAF}\\ & & \times \left(1-\mathrm{EAF}\right)+2\times SE^{2}\times N\times \mathrm{EAF}\times (1=\beta^{2}/\left(\beta^{2}+SE^{2}\times N\right) \end{array}$$

where EAF represented the effect allele frequency, *β* was the effect size of a SNP on the exposure factor, and SE was the standard error.(Pierce, Ahsan, and Vanderweele 2011) The steps to calculate the F‐statistic are as follows: obtain the sample size (*N*), determine the number of IVs (*K*), extract the effect allele frequency (EAF) for each IV from the GWAS data, extract the effect size (*β*) from the GWAS data, calculate *R* ^2^, and finally substitute *N*, *K*, and *R* ^2^into the formula to obtain the F‐statistic.

### 2.3 Forward MR Analysis

Five commonly used MR analyses were utilized in this study to assess the link of causality between white‐matter structural connectivity and AD, including the inverse variance weighted (IVW) method, Mendelian randomization, Egger regression (MR‐Egger), simple mode, weighted median, and weighted mode. IVW employs the Wald ratio method to perform association analysis for individual SNPs first and then selects either a fixed‐effects model or a random‐effects model to conduct a meta‐analysis for summarizing the effects of multiple loci (Burgess, Butterworth, and Thompson 2013, and Thompson 2016). MR Egger was initially a method used in meta‐analysis literature to assess “publication bias.” Through MR Egger, it is possible to assess the impact of such invalid instrumental variables on the estimation of causal effects and attempt to correct the biases arising from them (Burgess and Thompson 2017). Simple mode is a statistical method or result presentation in MR analysis, commonly used for initial screening and comparison. Weighted median is a robust statistical method used to combine data from multiple genetic variants into a single causal estimate (Bowden et al. 2016). Lastly, weighted mode calculates the weighted average of the effect sizes corresponding to multiple instrumental variables and determines the most common value of these effect sizes to obtain the final causal effect estimates. Among these five MR analysis methods, the IVW does not take into account the presence of an intercept term and assumes that there is no pleiotropy in SNPs. The statistical power of the IVW is higher than that of other methods; therefore, our research primarily relies on IVW.

We carried out the screening of significant exposures through the following three steps. Firstly, we used an MR circle plot to visualize the *P*‐values obtained from five MR methods for all AD‐related white‐matter structural connections. Using *P* < 0.05 as the threshold, where *P*‐values less than 0.05 are considered statistically significant, these significant results are represented by red squares in the MR circle plot. As our study primarily relies on the IVW, we initially screened AD‐related white‐matter structural connectivity with *P*‐values less than 0.05 using IVW for subsequent assessment of OR value direction and sensitivity analysis. Secondly, these selected exposures need to be excluded when they do not satisfy that the directions of OR values obtained from these five MR methods are the same. Lastly, we ran the horizontal pleiotropy and heterogeneity tests to determine the most stable exposures. The Cochran's Q test was calculated to access the heterogeneity of SNPs, and when significant heterogeneity existed (*P* <0.05), they need to be rejected. When horizontal pleiotropy in IVs was tested using MR‐Egger regression, pleiotropy was shown by an intercept term *P*‐value of less than 0.05 (Bowden, Davey Smith, and Burgess 2015; Burgess and Thompson 2017). At last, to precisely locate potentially heterogeneous SNPs, we eliminated each IV individually in turn for “leave‐one‐out” analysis.

R software (version 4.3.3) was used to perform the statistical analyses. All data screening, analysis and visualization were done separately using the R packages “TwoSampleMR,” “ieugwasr,” “VariantAnnotation,” “gwasglue,” “reshape2,” “circlize,” “ComplexHeatmap,” “grid,” “readr,” and “forestploter.”

### 2.4 Reverse MR Analysis

In order to explore the changes in white‐matter structural connectivity after the onset of AD, we conducted a reverse MR analysis. We only performed reverse MR analysis on the white‐matter structural connectivity that had a significant causal relationship with AD in the forward analysis. A more stringent genome‐wide significance threshold was used (*P* < 5×10^−8^). Similar to the forward analysis, we set the clustering window size to 10,000 kb and *R* ^2^ < 0.001 to remove SNPs with LD. The specific analysis steps of the reverse MR analysis are the same as those of the forward MR analysis.

## 3 Results

### 3.1 Selection of IVs

In our investigation, we identified 4087 SNPs with white‐matter structural connections using a genome‐wide significance threshold of *P* < 5×10^−6^ to make sure the requirements satisfied the MR assumption. The F‐statistics for the SNPs ranged from 20.8362 to 184.1775, which were all over 10, implying that these IVs lacked weak instrumental bias (Table S1).

### 3.2 Forward MR Analysis

**FIGURE 2:**
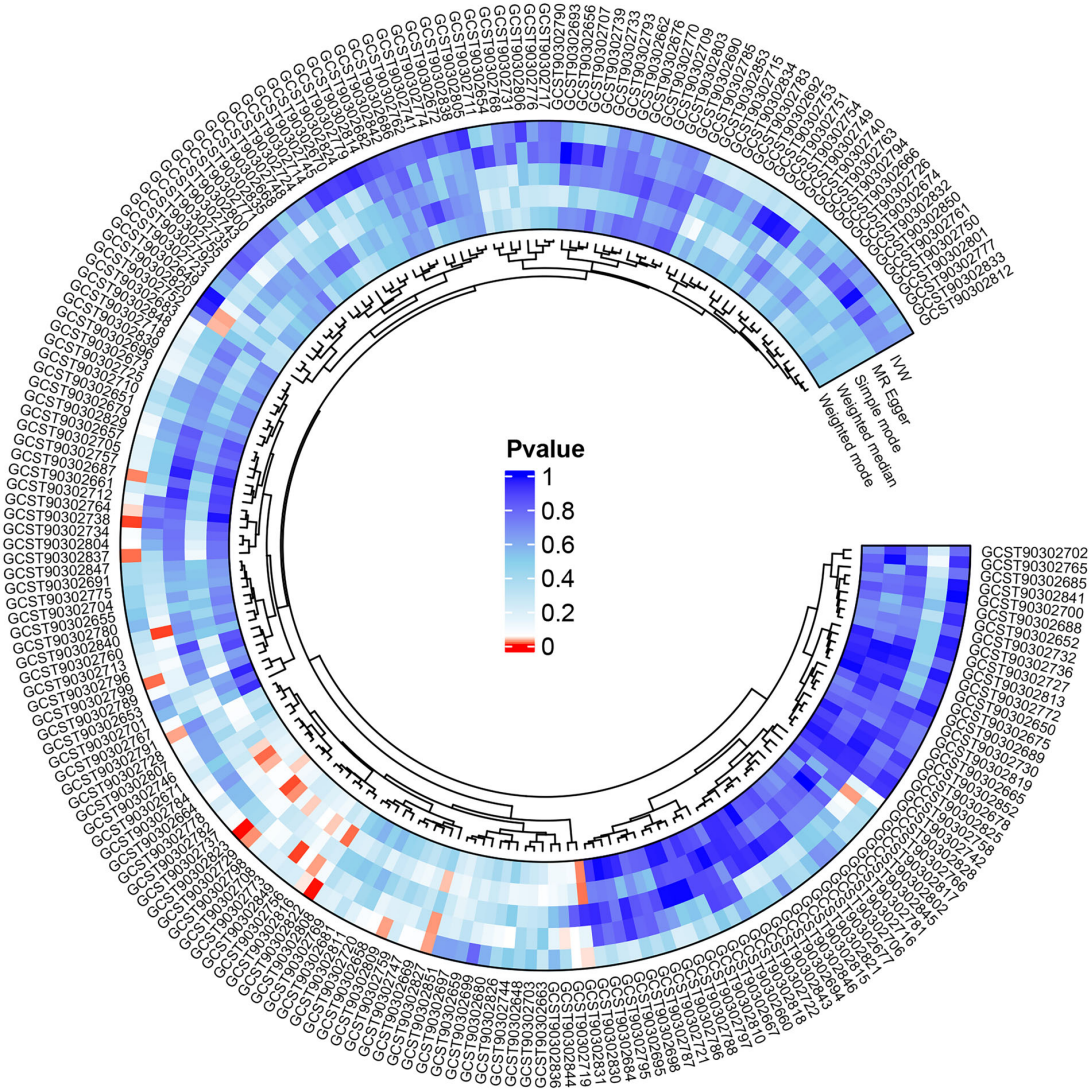
MR circle plot for the causal relationship of white‐matter structural connectivity on AD. The outermost GCST code encompasses all 206 structural connectivity measures. The blue circles, from the outer to the inner, represent different MR analysis methods: IVW, MR Egger, simple mode, weighted median, and weighted mode. Exposure factors with *p*‐value of less than 0.05 (red labels) obtained by the IVW method (outermost circle).

As shown in Figure 2, 13 white‐matter structural connections were significantly associated with the heritable susceptibility to AD based on the initial screening of the IVW method (*P* < 0.05), specifically including Right‐hemisphere salience_ventral attention network to accumbens white‐matter structural connectivity (OR: 1.24; 95% CI: 1.11–1.40; *P* = 0.0003), Right‐hemisphere salience_ventral attention network to amygdala white‐matter structural connectivity (OR: 0.79; 95% CI: 0.69–0.90; *P* = 0.0006), Left‐hemisphere limbic network to putamen white‐matter structural connectivity (OR: 0.86; 95% CI: 0.78–0.96; *P* = 0.0057), Right‐hemisphere somatomotor network to amygdala white‐matter structural connectivity (OR: 0.88; 95% CI: 0.80–0.98; *P* = 0.0137), Right‐hemisphere control network to caudate white‐matter structural connectivity (OR: 0.83; 95% CI: 0.71–0.96; *P* = 0.0141), Left‐hemisphere somatomotor network to amygdala white‐matter structural connectivity (OR: 0.87; 95% CI: 0.77–0.98; *P* = 0.0189), Left‐hemisphere limbic network to right‐hemisphere visual network white‐matter structural connectivity (OR: 0.84; 95% CI: 0.72–0.98; *P* = 0.0218), Right‐hemisphere dorsal attention network to pallidum white‐matter structural connectivity (OR: 1.15; 95% CI: 1.02–1.29; *P* = 0.0257), Right‐hemisphere limbic network to thalamus white‐matter structural connectivity (OR: 1.11; 95% CI: 1.01–1.23; *P* = 0.0260), Right‐hemisphere somatomotor network to right‐hemisphere limbic network white‐matter structural connectivity (OR: 0.82; 95% CI: 0.69–0.98; *P* = 0.0268), Left‐hemisphere default mode network to right‐hemisphere limbic network white‐matter structural connectivity (OR: 1.12; 95% CI: 1.01–1.25; *P* = 0.0393), Right‐hemisphere limbic network to hippocampus white‐matter structural connectivity (OR: 0.89; 95% CI: 0.80–1.00; *P* = 0.0422) and Right‐hemisphere salience_ventral attention network to right‐hemisphere default mode network white‐matter structural connectivity (OR: 0.91; 95% CI: 0.82–1.00; *P* = 0.0439) (Table S2). In MR analysis, the consistency of OR value directions is very important. When the directions of OR values obtained from multiple MR methods are inconsistent, it suggests the presence of unknown confounding factors. Taking a longitudinal look at the OR values of these 13 exposure factors obtained by the five MR methods, we found that the Left‐hemisphere default mode network to the right‐hemisphere limbic network white‐matter structural connectivity, Right‐hemisphere dorsal attention network to the pallidum white‐matter structural connectivity, and the Right‐hemisphere control network to caudate white‐matter structural connectivity did not satisfy that their OR values were all greater than 1 or all less than 1, and thus these 3 white‐matter structural connections needed to be eliminated (Table 1).

**TABLE 1:** The OR values of 13 white‐matter structural connections significantly associated with AD genetic susceptibility obtained through five MR methods.

Id.exposure	Outcome	Exposure	Method	Or
GCST90302687	Alzheimer disease	Left‐hemisphere somatomotor network to amygdala white‐matter structural connectivity	MR Egger	0.848305
GCST90302687	Alzheimer disease	Left‐hemisphere somatomotor network to amygdala white‐matter structural connectivity	Weighted median	0.939446
GCST90302687	Alzheimer disease	Left‐hemisphere somatomotor network to amygdala white‐matter structural connectivity	Inverse variance weighted	0.869358
GCST90302687	Alzheimer disease	Left‐hemisphere somatomotor network to amygdala white‐matter structural connectivity	Simple mode	0.966649
GCST90302687	Alzheimer disease	Left‐hemisphere somatomotor network to amygdala white‐matter structural connectivity	Weighted mode	0.974522
GCST90302729	Alzheimer disease	Left‐hemisphere limbic network to right‐hemisphere visual network white‐matter structural connectivity	MR Egger	0.755415
GCST90302729	Alzheimer disease	Left‐hemisphere limbic network to right‐hemisphere visual network white‐matter structural connectivity	Weighted median	0.817282
GCST90302729	Alzheimer disease	Left‐hemisphere limbic network to right‐hemisphere visual network white‐matter structural connectivity	Inverse variance weighted	0.840213
GCST90302729	Alzheimer disease	Left‐hemisphere limbic network to right‐hemisphere visual network white‐matter structural connectivity	Simple mode	0.755179
GCST90302729	Alzheimer disease	Left‐hemisphere limbic network to right‐hemisphere visual network white‐matter structural connectivity	Weighted mode	0.795679
GCST90302738	Alzheimer disease	Left‐hemisphere limbic network to putamen white‐matter structural connectivity	MR Egger	0.960882
GCST90302738	Alzheimer disease	Left‐hemisphere limbic network to putamen white‐matter structural connectivity	Weighted median	0.916167
GCST90302738	Alzheimer disease	Left‐hemisphere limbic network to putamen white‐matter structural connectivity	Inverse variance weighted	0.861662
GCST90302738	Alzheimer disease	Left‐hemisphere limbic network to putamen white‐matter structural connectivity	Simple mode	0.951147
GCST90302738	Alzheimer disease	Left‐hemisphere limbic network to putamen white‐matter structural connectivity	Weighted mode	0.9735
GCST90302764^*^	Alzheimer disease	Left‐hemisphere default mode network to right‐hemisphere limbic network white‐matter structural connectivity	MR Egger	0.949611
GCST90302764^*^	Alzheimer disease	Left‐hemisphere default mode network to right‐hemisphere limbic network white‐matter structural connectivity	Weighted median	1.065026
GCST90302764^*^	Alzheimer disease	Left‐hemisphere default mode network to right‐hemisphere limbic network white‐matter structural connectivity	Inverse variance weighted	1.123457
GCST90302764^*^	Alzheimer disease	Left‐hemisphere default mode network to right‐hemisphere limbic network white‐matter structural connectivity	Simple mode	1.034236
GCST90302764^*^	Alzheimer disease	Left‐hemisphere default mode network to right‐hemisphere limbic network white‐matter structural connectivity	Weighted mode	1.002549
GCST90302791	Alzheimer disease	Right‐hemisphere somatomotor network to right‐hemisphere limbic network white‐matter structural connectivity	MR Egger	0.904599
GCST90302791	Alzheimer disease	Right‐hemisphere somatomotor network to right‐hemisphere limbic network white‐matter structural connectivity	Weighted median	0.835766
GCST90302791	Alzheimer disease	Right‐hemisphere somatomotor network to right‐hemisphere limbic network white‐matter structural connectivity	Inverse variance weighted	0.821641
GCST90302791	Alzheimer disease	Right‐hemisphere somatomotor network to right‐hemisphere limbic network white‐matter structural connectivity	Simple mode	0.830695
GCST90302791	Alzheimer disease	Right‐hemisphere somatomotor network to right‐hemisphere limbic network white‐matter structural connectivity	Weighted mode	0.83982
GCST90302799	Alzheimer disease	Right‐hemisphere somatomotor network to amygdala white‐matter structural connectivity	MR Egger	0.831426
GCST90302799	Alzheimer disease	Right‐hemisphere somatomotor network to amygdala white‐matter structural connectivity	Weighted median	0.929536
GCST90302799	Alzheimer disease	Right‐hemisphere somatomotor network to amygdala white‐matter structural connectivity	Inverse variance weighted	0.884779
GCST90302799	Alzheimer disease	Right‐hemisphere somatomotor network to amygdala white‐matter structural connectivity	Simple mode	0.950721
GCST90302799	Alzheimer disease	Right‐hemisphere somatomotor network to amygdala white‐matter structural connectivity	Weighted mode	0.939676
GCST90302809^*^	Alzheimer disease	Right‐hemisphere dorsal attention network to pallidum white‐matter structural connectivity	MR Egger	0.806234
GCST90302809^*^	Alzheimer disease	Right‐hemisphere dorsal attention network to pallidum white‐matter structural connectivity	Weighted median	1.097739
GCST90302809^*^	Alzheimer disease	Right‐hemisphere dorsal attention network to pallidum white‐matter structural connectivity	Inverse variance weighted	1.146269
GCST90302809^*^	Alzheimer disease	Right‐hemisphere dorsal attention network to pallidum white‐matter structural connectivity	Simple mode	1.153282
GCST90302809^*^	Alzheimer disease	Right‐hemisphere dorsal attention network to pallidum white‐matter structural connectivity	Weighted mode	1.111253
GCST90302816	Alzheimer disease	Right‐hemisphere salience_ventral attention network to right‐hemisphere default mode network white‐matter structural connectivity	MR Egger	0.74247
GCST90302816	Alzheimer disease	Right‐hemisphere salience_ventral attention network to right‐hemisphere default mode network white‐matter structural connectivity	Weighted median	0.886502
GCST90302816	Alzheimer disease	Right‐hemisphere salience_ventral attention network to right‐hemisphere default mode network white‐matter structural connectivity	Inverse variance weighted	0.905374
GCST90302816	Alzheimer disease	Right‐hemisphere salience_ventral attention network to right‐hemisphere default mode network white‐matter structural connectivity	Simple mode	0.842938
GCST90302816	Alzheimer disease	Right‐hemisphere salience_ventral attention network to right‐hemisphere default mode network white‐matter structural connectivity	Weighted mode	0.856872
GCST90302822	Alzheimer disease	Right‐hemisphere salience_ventral attention network to amygdala white‐matter structural connectivity	MR Egger	0.714695
GCST90302822	Alzheimer disease	Right‐hemisphere salience_ventral attention network to amygdala white‐matter structural connectivity	Weighted median	0.812288
GCST90302822	Alzheimer disease	Right‐hemisphere salience_ventral attention network to amygdala white‐matter structural connectivity	Inverse variance weighted	0.790206
GCST90302822	Alzheimer disease	Right‐hemisphere salience_ventral attention network to amygdala white‐matter structural connectivity	Simple mode	0.821285
GCST90302822	Alzheimer disease	Right‐hemisphere salience_ventral attention network to amygdala white‐matter structural connectivity	Weighted mode	0.824288
GCST90302823	Alzheimer disease	Right‐hemisphere salience_ventral attention network to accumbens white‐matter structural connectivity	MR Egger	1.14786
GCST90302823	Alzheimer disease	Right‐hemisphere salience_ventral attention network to accumbens white‐matter structural connectivity	Weighted median	1.245514
GCST90302823	Alzheimer disease	Right‐hemisphere salience_ventral attention network to accumbens white‐matter structural connectivity	Inverse variance weighted	1.244661
GCST90302823	Alzheimer disease	Right‐hemisphere salience_ventral attention network to accumbens white‐matter structural connectivity	Simple mode	1.297774
GCST90302823	Alzheimer disease	Right‐hemisphere salience_ventral attention network to accumbens white‐matter structural connectivity	Weighted mode	1.2727
GCST90302827	Alzheimer disease	Right‐hemisphere limbic network to thalamus white‐matter structural connectivity	MR Egger	1.305339
GCST90302827	Alzheimer disease	Right‐hemisphere limbic network to thalamus white‐matter structural connectivity	Weighted median	1.137259
GCST90302827	Alzheimer disease	Right‐hemisphere limbic network to thalamus white‐matter structural connectivity	Inverse variance weighted	1.114327
GCST90302827	Alzheimer disease	Right‐hemisphere limbic network to thalamus white‐matter structural connectivity	Simple mode	1.100701
GCST90302827	Alzheimer disease	Right‐hemisphere limbic network to thalamus white‐matter structural connectivity	Weighted mode	1.096709
GCST90302831	Alzheimer disease	Right‐hemisphere limbic network to hippocampus white‐matter structural connectivity	MR Egger	0.845693
GCST90302831	Alzheimer disease	Right‐hemisphere limbic network to hippocampus white‐matter structural connectivity	Weighted median	0.853532
GCST90302831	Alzheimer disease	Right‐hemisphere limbic network to hippocampus white‐matter structural connectivity	Inverse variance weighted	0.890241
GCST90302831	Alzheimer disease	Right‐hemisphere limbic network to hippocampus white‐matter structural connectivity	Simple mode	0.987509
GCST90302831	Alzheimer disease	Right‐hemisphere limbic network to hippocampus white‐matter structural connectivity	Weighted mode	0.817801
GCST90302837^*^	Alzheimer disease	Right‐hemisphere control network to caudate white‐matter structural connectivity	MR Egger	1.197847
GCST90302837^*^	Alzheimer disease	Right‐hemisphere control network to caudate white‐matter structural connectivity	Weighted median	0.868581
GCST90302837^*^	Alzheimer disease	Right‐hemisphere control network to caudate white‐matter structural connectivity	Inverse variance weighted	0.82873
GCST90302837^*^	Alzheimer disease	Right‐hemisphere control network to caudate white‐matter structural connectivity	Simple mode	0.94668
GCST90302837^*^	Alzheimer disease	Right‐hemisphere control network to caudate white‐matter structural connectivity	Weighted mode	0.957628

*Note*: The directions of OR values for GCST90302764, GCST90302809, and GCST90302837 were inconsistent across the five MR methods. These three codes have been bolded and marked with an asterisk in the top right corner, and they were not included in the subsequent MR analysis.

**FIGURE 3:**
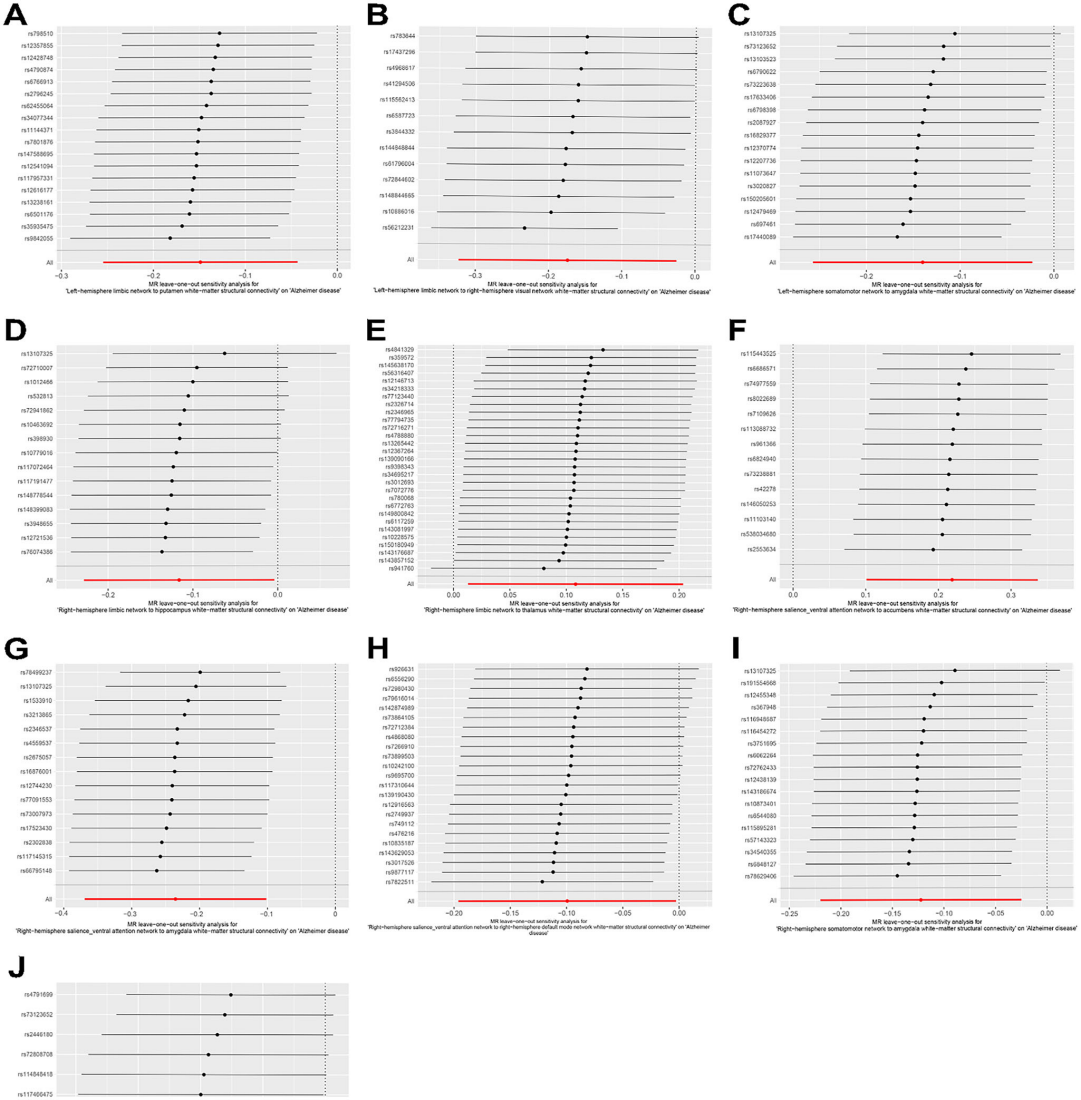
MR “leave‐one‐out” plots for the causal relationship of white‐matter structural connectivity on AD. From A to J, the MR effects of the remaining IVs after excluding these SNPs were consistent with the overall trend of the MR effect, and no SNPs with significant heterogeneity were found. (A) Left‐hemisphere limbic network to putamen white‐matter structural connectivity. (B) Left‐hemisphere limbic network to right‐hemisphere visual network white‐matter structural connectivity. (C) Left‐hemisphere somatomotor network to amygdala white‐matter structural connectivity. (D) Right‐hemisphere limbic network to hippocampus white‐matter structural connectivity. (E) Right‐hemisphere limbic network to thalamus white‐matter structural connectivity. (F) Right‐hemisphere salience_ventral attention network to accumbens white‐matter structural connectivity. (G) Right‐hemisphere salience_ventral attention network to amygdala white‐matter structural connectivity. (H) Right‐hemisphere salience_ventral attention network to right‐hemisphere default mode network white‐matter structural connectivity. (I) Right‐hemisphere somatomotor network to amygdala white‐matter structural connectivity. (J) Right‐hemisphere somatomotor network to right‐hemisphere limbic network white‐matter structural connectivity.

Subsequently, in a sensitivity analysis, the IVs of Right‐hemisphere control network to caudate white‐matter structural connectivity showed significant heterogeneity in Cochran's Q test for IVW and MR Egger (*P* < 0.05) (Table S3). MR‐Egger regression demonstrated the presence of horizontal pleiotropy of Right‐hemisphere dorsal attention network to pallidum white‐matter structural connectivity (*P* < 0.05) (Table S4). However, the “leave‐one‐out” analysis did not reveal any SNPs with considerable heterogeneity, indicating that eliminating any SNP will not alter the general pattern of AD incidence (Figure 3). Through multiple filters and screening, we ended up with 10 white‐matter structural connections strongly linked with AD pathogenesis.

**FIGURE 4:**
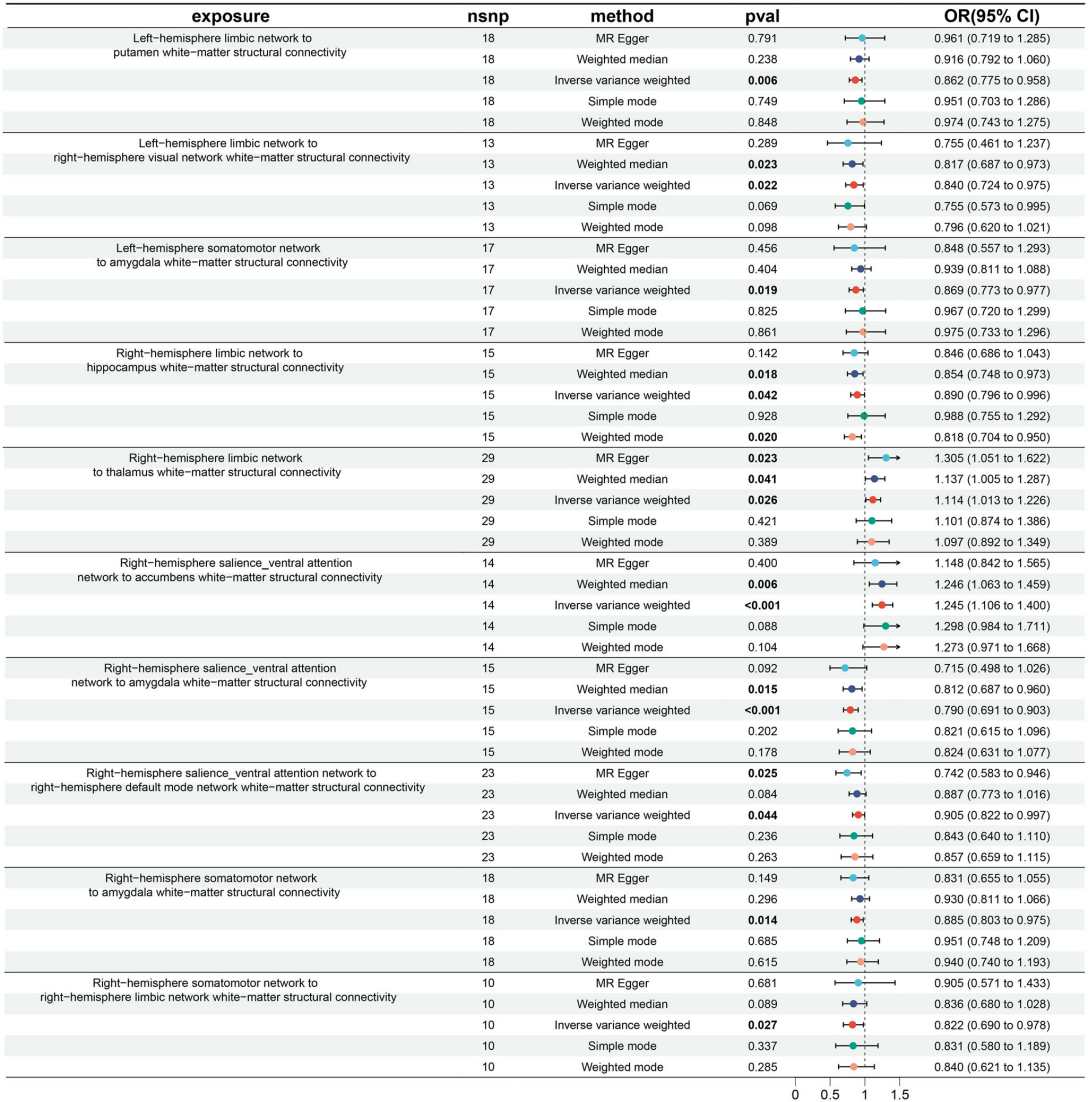
MR forest plots for the causal relationship of white‐matter structural connectivity on AD. The OR values obtained by the IVW for all 10 connections were statistically significant (*P* < 0.05, in bold). Among these 10 connections, the OR values for Right‐hemisphere limbic network to thalamus white‐matter structural connectivity and Right‐hemisphere salience_ventral attention network to accumbens white‐matter structural connectivity are greater than 1 in all five MR methods. The OR values for the remaining 8 connections are all less than 1 in all five MR methods.

**FIGURE 5:**
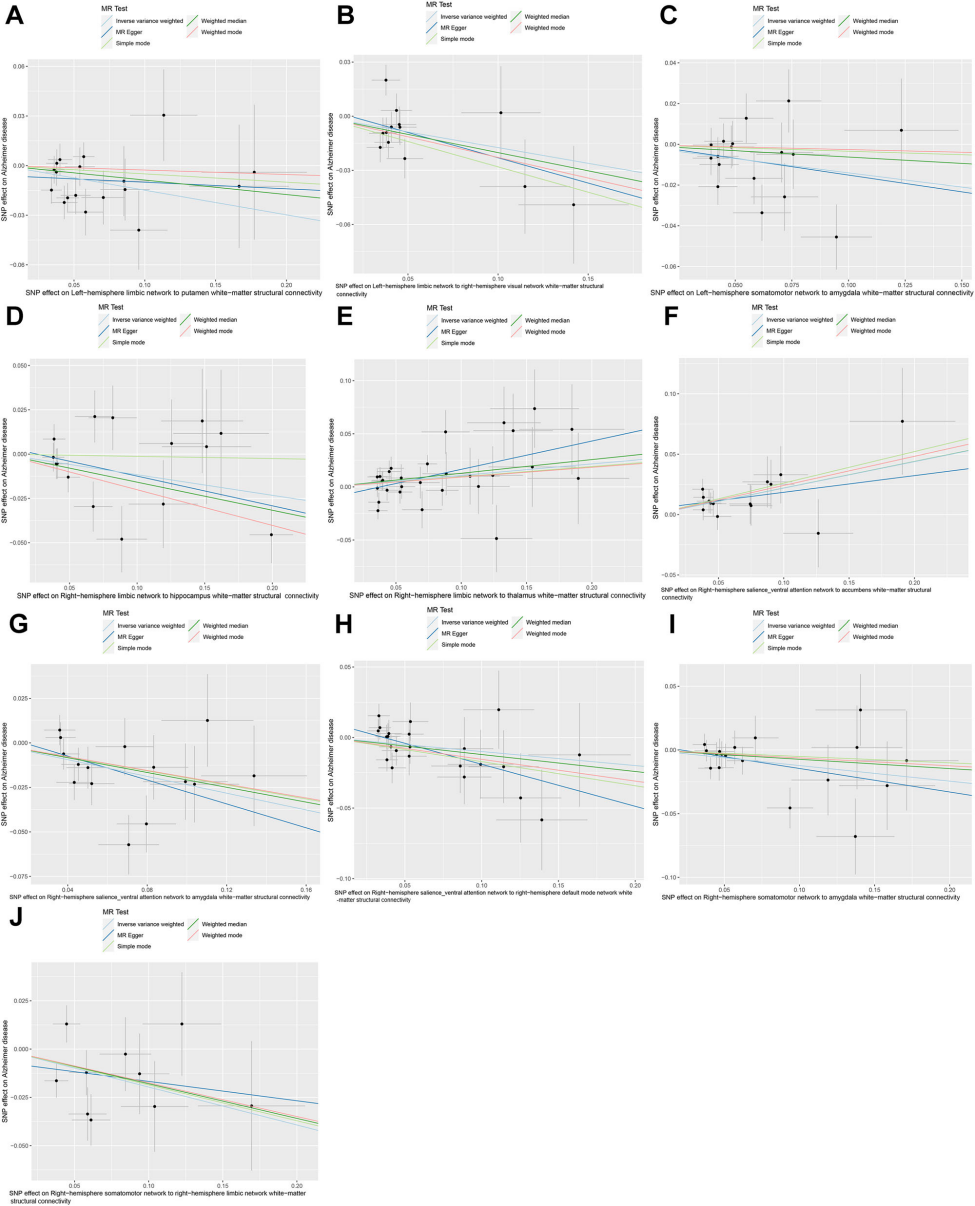
MR scatter plots for the causal relationship of white‐matter structural connectivity on AD. Among these 10 connections, the scatter plot slopes for the five MR methods of the Right‐hemisphere limbic network to thalamus white‐matter structural connectivity and the Right‐hemisphere salience_ventral attention network to accumbens white‐matter structural connectivity show an upward trend. The scatter plot slopes for the five MR methods of the remaining 8 connections show a downward trend. (A) Left‐hemisphere limbic network to putamen white‐matter structural connectivity. (B) Left‐hemisphere limbic network to right‐hemisphere visual network white‐matter structural connectivity. (C) Left‐hemisphere somatomotor network to amygdala white‐matter structural connectivity. (D) Right‐hemisphere limbic network to hippocampus white‐matter structural connectivity. (E) Right‐hemisphere limbic network to thalamus white‐matter structural connectivity. (F) Right‐hemisphere salience_ventral attention network to accumbens white‐matter structural connectivity. (G) Right‐hemisphere salience_ventral attention network to amygdala white‐matter structural connectivity. (H) Right‐hemisphere salience_ventral attention network to right‐hemisphere default mode network white‐matter structural connectivity. (I) Right‐hemisphere somatomotor network to amygdala white‐matter structural connectivity. (J) Right‐hemisphere somatomotor network to right‐hemisphere limbic network white‐matter structural connectivity.

The MR analysis results might be visualized using scatter plots and forest plots. The OR values of Right‐hemisphere limbic network to thalamus white‐matter structural connectivity and Right‐hemisphere salience_ventral attention network to accumbens white‐matter structural connectivity were both greater than 1, suggesting that the more of these two connections, the higher the risk of developing AD (Figure 4). In contrast, the remaining eight connections were negatively associated with the risk of AD. Similarly, we more intuitively found that the SNP effect on AD was progressively increasing with SNP effects on Right‐hemisphere limbic network to thalamus white‐matter structural connectivity and right‐hemisphere salience_ventral attention network to accumbens white‐matter structural connectivity using the scatterplot in Figure 5E,F.

### 3.3 Reverse MR Analysis

We conducted a reverse MR analysis with AD as the exposure and specific white‐matter structural connectivity as the outcome, but no significant reverse causal relationship was found between them (*P* > 0.05) (Table S5). Based on the Cochran's Q test for IVW and MR‐Egger, significant heterogeneity was observed in the IVs for Left‐hemisphere limbic network to right‐hemisphere visual network white‐matter structural connectivity, Right‐hemisphere limbic network to thalamus white‐matter structural connectivity, Right‐hemisphere salience_ventral attention network to accumbens white‐matter structural connectivity, Right‐hemisphere salience_ventral attention network to amygdala white‐matter structural connectivity, and Right‐hemisphere somatomotor network to right‐hemisphere limbic network white‐matter structural connectivity when AD was the exposure factor (*P* < 0.05) (Table S6). MR‐Egger regression indicated the presence of horizontal pleiotropy for Left‐hemisphere limbic network to right‐hemisphere visual network white‐matter structural connectivity, Right‐hemisphere salience_ventral attention network to amygdala white‐matter structural connectivity, and Right‐hemisphere salience_ventral attention network to right‐hemisphere default mode network white‐matter structural connectivity (*P* < 0.05) (Table S7). The “leave‐one‐out” sensitivity analysis did not identify any SNPs with significant heterogeneity, indicating that the risk estimates associated with AD and specific white‐matter structural connectivity cannot be attributed to a single SNP (Figure S1).

## 4 Discussion

### 4.1 Overview

This study used a two‐sample MR technique to evaluate the most recent summary GWAS data and revealed a causal relationship between white‐matter structural connectivity and AD, which is the first time that the relationship between these two has been investigated at the genetic level. We obtained over 4000 strong IVs through initial screening using a genome‐wide significance threshold of *P* < 5×10^−6^ (Table S1). Subsequently, we screened for AD‐related white‐matter structural connectivity (Table S2). Through OR value direction, heterogeneity, and horizontal pleiotropy, we ultimately identified 10 white‐matter connections that we needed, including Left‐hemisphere limbic network to putamen white‐matter structural connectivity, Left‐hemisphere limbic network to right‐hemisphere visual network white‐matter structural connectivity, Left‐hemisphere somatomotor network to amygdala white‐matter structural connectivity, Right‐hemisphere limbic network to hippocampus white‐matter structural connectivity, Right‐hemisphere salience_ventral attention network to amygdala white‐matter structural connectivity, Right‐hemisphere salience_ventral attention network to right‐hemisphere default mode network white‐matter structural connectivity, Right‐hemisphere somatomotor network to amygdala white‐matter structural connectivity and Right‐hemisphere somatomotor network to right‐hemisphere limbic network white‐matter structural connectivity are protective factors for AD, while Right‐hemisphere limbic network to thalamus white‐matter structural connectivity and Right‐hemisphere salience_ventral attention network to accumbens white‐matter structural connectivity have adverse effects on AD (Table 1; Tables S3 and S4).

Collider bias is a particular issue that needs attention in MR analysis. It refers to the fact that when two or more exposure factors combine to influence an intermediate variable, this intermediate variable may become a confounder distorting the true association between exposure and outcome (Coscia et al. 2022). In our study, the analyses we obtained through hypothesis testing, screening for exposures and outcomes, screening for IVs, heterogeneity assessment, horizontal pleiotropy testing, sensitivity analyses, and visualization of results were robust and accurate. Such comprehensive analysis steps can effectively reduce collider bias in MR analysis and enhance the reliability of the results. Such results mean that white‐matter structural connectivity is expected to be a reliable predictor of AD and contribute to the medical management of AD patients.

In the reverse MR analysis, all 10 AD‐related white‐matter structural connections had *P*‐values greater than 0.05, meaning that these 10 white‐matter structural connections that was positive in the forward MR analysis were not statistically significant in the reverse MR analysis. Therefore, in the first step of the reverse MR analysis, we found that there is no significant reverse causality between AD and specific white‐matter structural connections. Subsequently, the IVs of multiple white‐matter structural connectivity exhibit heterogeneity and pleiotropy. Heterogeneity may lead to biased or unstable results in the reverse analysis. Pleiotropy may lead to complex and difficult‐to‐interpret relationships between genetic variants and outcomes. The factors with heterogeneity and pleiotropy should be excluded. We may believe that the white‐matter structural connectivity that were positive in the forward analysis do not have an effect on the onset or progression of AD based on the results of all steps in the reverse MR analysis (Tables S5–S7 and Figure S1).

### 4.2 Positive White‐Matter Structural Connectivity for AD

Interactions between different brain regions sustain the human brain's daily functioning. Genetic variation constantly affects the structural connectivity of these brain regions, which produces corresponding functional changes. Elsheikh et al. identified significant SNPs and genes by quantifying changes in the brain connectome of AD patients from a global brain perspective.(Elsheikh et al. 2020). Wainberg et al. used genetic covariance analyzer method to calculate genetic correlations between 206 GWAS and 15 powerful GWAS for cognitive traits. (Wainberg et al. 2024) These connections can be classified into hemisphere‐level cortical‐to‐cortical connectivity, network‐level cortical‐to‐cortical connectivity within and between each of the “Yeo 7” networks and cortical‐to‐subcortical connectivity between each of the “Yeo 7” networks and 7 subcortical structures. Our study identified 10 white‐matter connections that have a causal relationship with AD, including 7 cortical‐to‐subcortical connections and 3 cortical‐to‐cortical connections between different “Yeo 7” networks. Increased structural connectivity in brain regions was isotropic to increased risk of AD when looking across the whole genome (Wainberg et al. 2024). However, this is partially inconsistent with the results of this article. The atrophy of intracranial structures in AD patients is mainly reflected in the hippocampus, medial temporal lobe and amygdala, whereas Left‐hemisphere somatomotor network to amygdala white‐matter structural connectivity, Right‐hemisphere limbic network to hippocampus white‐matter structural connectivity, Right‐hemisphere salience_ventral attention network to amygdala white‐matter structural connectivity and Right‐hemisphere somatomotor network to amygdala white‐matter structural connectivity are all protective factors for AD, which probably indicates that when cortical networks are more structurally connected to the amygdala or hippocampus, this can lead to higher hippocampal and amygdaloid mobility and reduced atrophy, which in turn slows down the development of AD. Kim et al. found enhanced hippocampal‐inferotemporal gyrus white matter connectivity and remission of disease progression in their early AD patients after pharmacologic treatment, which is similar to our findings (Kim et al. 2023).

The medial temporal lobe belongs to the limbic system of the brain, which is closely related to memory function and dominates in the pathogenesis of AD (Squire, Stark, and Clark 2004). Other clinical manifestations of AD patients include aphasia, dysarthria, visuospatial impairment, and behavioral changes (Zhang et al. 2023). Obviously, these abnormal functional changes are closely related to the structure of the limbic network, visual network, attention network, and motor network. Elevated coupling of Left‐hemisphere limbic network to putamen white‐matter structural connectivity, Left‐hemisphere limbic network to right‐hemisphere visual network white‐matter structural connectivity, Right‐hemisphere salience_ventral attention network to right‐hemisphere default mode network white‐matter structural connectivity, and Right‐hemisphere somatomotor network to right‐hemisphere limbic network white‐matter structural connectivity increased the interaction between these four networks, reduced functional disruption, and ameliorated cognitive impairment and behavioral damage, so they also became protective factors for AD. Although there are currently no similar studies indicating that enhanced connectivity of the medial temporal lobe, hippocampus, or amygdala can prevent AD, numerous studies have found that the widespread decrease in connectivity in these regions among AD patients indirectly confirms the conclusions of our research (King‐Robson, Wilson, and Politis 2021; Ortner et al. 2016).

### 4.3 Negative White‐Matter Structural Connectivity for AD

AD is associated with neuronal loss in the thalamus, with the thalamic nuclei being the primary sites of degeneration in AD (Zarei et al. 2010). Various studies have shown the involvement of corticothalamic networks in the regulation of cognitive and behavioral aspects of AD, including attention, sleep maintenance, cognition and memory (Alescio‐Lautier et al. 2007; Benedict et al. 2015; Bracco et al. 2014; Jagirdar and Chin 2019; Westwood et al. 2017). Through certain synapses, cortical networks and thalamic neurons can interact to form cortico‐thalamo‐cortical (CTC) loops. The CTC loops play the role in sensory, motor and cognitive domains, making corticothalamic connections more important than other types of connections, and they are more heritable and susceptible to specific genomic control (Shepherd and Yamawaki 2021; Wainberg et al. 2024). The control of these processes is largely dependent on the thalamic reticular nucleus (TRN). Anderson et al. and Beenhakker et al. found that TRN was particularly active during sleep, which inhibited the transmission of sensory information between thalamic relay neurons and the cortex (Anderson et al. 2005; Beenhakker and Huguenard 2009). Wells et al. suggested that TRN‐driven dynamic inhibition of thalamic relay neurons was the key to focused attention (Wells et al. 2016). Therefore, when Right‐hemisphere limbic network to thalamus white‐matter structural connectivity became a risk factor for AD, the increased connectivity between the cortical network and the thalamus led to dysregulation of TRN activity, inhibiting cognitive abilities in AD patients.

A crucial component of the brain's reward system, the nucleus accumbens is involved in feelings of pleasure, addiction, aggression, fear, and the placebo effect (Volkow and Morales 2015). The medium spiny neurons (MSNs) are the fundamental cell type of the nucleus accumbens, which generate the inhibitory neurotransmitter, gamma‐aminobutyric acid (GABA) (Floresco 2015). It has been shown that Aβ accumulates in the limbic system, affecting the functioning of the accumbens and altering cognitive and emotional behaviors (Brilliant et al. 1997; Pievani et al. 2013). Fernández‐Pérez et al. found that Aβ deposits were present in the nucleus accumbens of AD mice, which led to increased excitability of MSNs, active synaptic transmission, and inhibition of dopamine secretion, resulting in anhedonia and emotional indifference (Fernández‐Pérez et al. 2020). When the connections between the attention network and the accumbens increase, the excitability of MSN enhances, releasing GABA to affect attention, which perhaps also explains why Right‐hemisphere salience_ventral attention network to accumbens white‐matter structural connectivity becomes another risk factor for the onset of AD.

### 4.4 Advantages and Limitations

Our study is the first to use MR analysis to investigate the association between AD and white‐matter structural connectivity using the most extensive GWAS database. Based on our results, we can provide hypothetical and targeted suggestions for AD patients. Early fiber tractography can be performed in patients with mild cognitive impairment to see how active the Right‐hemisphere limbic network to thalamus white‐matter structural connectivity and Right‐hemisphere salience_ventral attention network to accumbens white‐matter structural connectivity are. The early detection of such AD risk factors may help in formulating effective intervention measures ahead of time, as firm conclusions require actual data as a foundation.

But there are still certain restrictions on this study. Firstly, regarding the study's participants, different centers may have different diagnostic criteria for AD. If patients in the pre‐dementia stage of AD or other types of dementia are included in the study, the authenticity of the results obtained will be worth examining. Moreover, the GWAS data for both white‐matter structural connectivity and AD were derived from European populations, which may lead to bias when analyzing the population at baseline. In the future, other ethnic groups need to be included depending on the specific research content (Hirata et al. 2017; Walters et al. 2023). In recent years, some studies have linked white matter to AD pathological products or mechanistic pathways at the microscopic and molecular level of AD pathology (Lorenzini et al. 2024; Tranfa et al. 2024). In the future, early identification of pertinent white‐matter structural connectivity and molecular pathways may help reduce AD pathological products and aid in AD treatment. However, our research has not yet delved into the molecular level, which represents a significant gap. In the future, we will consider incorporating relevant mechanistic studies.

## 5 Conclusion

In conclusion, our two‐sample MR investigation established a causal relationship between the onset of AD and some specific white‐matter structural connections. Right‐hemisphere limbic network to thalamus white‐matter structural connectivity and Right‐hemisphere salience_ventral attention network to accumbens white‐matter structural connectivity were risk factors for AD, whereas the remaining 8 connections had protective effects against AD. There is no reverse causal relationship between AD and these 10 white‐matter structural connections. This provides some support for the idea that the variety of white‐matter structural connectivity may be utilized to diagnose, treat, and prevent AD. It also offers a fresh perspective and justification for further AD research.

## Author Contributions

**Siyu Liu**: methodology, investigation and writing–original draft. **Daoying Geng**: supervision, writing–review, and editing.

## Conflicts of Interest

The authors declare no conflicts of interest.

### Peer Review

The peer review history for this article is available at https://publons.com/publon/10.1002/brb3.70286.

## Supporting information



Supplementary Table S1: The F‐statistics for all SNPs in forward MR analysis.

Supplementary Table S2: Relationship between 206 white‐matter structural connectivity and genetic susceptibility to AD based on the IVW method in forward MR analysis.

Supplementary Table S3: The heterogeneity test in Cochran's Q test for IVW and MR Egger in forward MR analysis.

Supplementary Table S4: The horizontal pleiotropy test by MR Egger regression in forward MR analysis

Supplementary Table S5: Relationship between AD and specific white‐matter structural connectivity based on the IVW method in reverse MR analysis.

Supplementary Table S6: The heterogeneity test in Cochran's Q test for IVW and MR Egger in reverse MR analysis.

Supplementary Table S7: The horizontal pleiotropy test by MR Egger regression in reverse MR analysis.

Supplementary Figure S1: Reverse MR "leave‐one‐out" plots for the causal relationship of AD on specific white‐matter structural connectivity.

## Data Availability

The datasets that are publicly available were used to examine the data for this study. The GWAS summary data of 206 white‐matter structural connections can be acquired from the European Bioinformatics Institute GWAS Catalog (data link: https://www.ebi.ac.uk/gwas; accession numbers: GCST90302648‐GCST90302853; https://doi.org/10.1038/s41467‐024‐46023‐2). AD GWAS data can be obtained from the European Bioinformatics Institute GWAS Catalog (data link: https://gwas.mrcieu.ac.uk/datasets/ebi‐a‐GCST90027158/; https://doi.org/10.1038/s41588‐022‐01024‐z).
